# Preferences of Knowledge Users for Two Formats of Summarizing Results from Systematic Reviews: Infographics and Critical Appraisals

**DOI:** 10.1371/journal.pone.0140029

**Published:** 2015-10-14

**Authors:** Katelynn Crick, Lisa Hartling

**Affiliations:** 1 School of Public Health, University of Alberta, Edmonton, Canada; 2 Department of Pediatrics, Alberta Research Centre for Health Evidence, University of Alberta, Edmonton, Canada; Johns Hopkins Bloomberg School of Public Health, UNITED STATES

## Abstract

**Objectives:**

To examine and compare preferences of knowledge users for two different formats of summarizing results from systematic reviews: infographics and critical appraisals.

**Design:**

Cross-sectional.

**Setting:**

Annual members’ meeting of a Network of Centres of Excellence in Knowledge Mobilization called TREKK (Translating Emergency Knowledge for Kids). TREKK is a national network of researchers, clinicians, health consumers, and relevant organizations with the goal of mobilizing knowledge to improve emergency care for children.

**Participants:**

Members of the TREKK Network attending the annual meeting in October 2013.

**Outcome Measures:**

Overall preference for infographic vs. critical appraisal format. Members’ rating of each format on a 10-point Likert scale for clarity, comprehensibility, and aesthetic appeal. Members’ impressions of the appropriateness of the two formats for their professional role and for other audiences.

**Results:**

Among 64 attendees, 58 members provided feedback (91%). Overall, their preferred format was divided with 24/47 (51%) preferring the infographic to the critical appraisal. Preference varied by professional role, with 15/22 (68%) of physicians preferring the critical appraisal and 8/12 (67%) of nurses preferring the infographic. The critical appraisal was rated higher for clarity (mean 7.8 vs. 7.0; p = 0.03), while the infographic was rated higher for aesthetic appeal (mean 7.2 vs. 5.0; p<0.001). There was no difference between formats for comprehensibility (mean 7.6 critical appraisal vs. 7.1 infographic; p = 0.09). Respondents indicated the infographic would be most useful for patients and their caregivers, while the critical appraisal would be most useful for their professional roles.

**Conclusions:**

Infographics are considered more aesthetically appealing for summarizing evidence; however, critical appraisal formats are considered clearer and more comprehensible. Our findings show differences in terms of audience-specific preferences for presentation of research results. This study supports other research indicating that tools for knowledge dissemination and translation need to be targeted to specific end users’ preferences and needs.

## Introduction

Health services research has consistently found a failure to translate healthcare research results into practice and policy.[1] Despite the millions of dollars that are spent each year on health research, healthcare systems continue to fail to ensure that effective programs, services, and drugs get to all patients who need them.[1]

Limited time is a frequently cited barrier to the use of evidence in practice.[2–6] Systematic reviews are considered a cornerstone of knowledge translation as they provide a comprehensive synthesis of available evidence on a clinical question for decision makers in the healthcare system.[1,7] Although systematic reviews are methodologically rigorous, users have requested that reviews be translated into formats that are shorter and more easily understood by different audiences.[8] Knowledge users need knowledge translation tools that provide synopses of systematic reviews with key messages highlighted for quick reference.

Summarized evidence for clinicians exists in many different formats including structured abstracts and synopses published in secondary journals.[9] Journals and other resources (e.g., UpToDate) that provide high-level summaries to inform evidence-based practice have used critical appraisal formats extensively. Critical appraisal formats summarize the research evidence and provide information on its validity and applicability for knowledge users.[10]

Infographics have emerged recently as a novel method of graphically displaying information. Infographics are visual images such as charts or diagrams that are used to represent information and data.[11] Research has shown that visualizations, as opposed to text, are intrinsically more memorable and effectively transferred with consistency across people.[12–14] High memorability scores are correlated with visualizations, including infographics, that contain pictograms, more color, low data-to-ink ratios, and high visual densities.[12,14] Infographics therefore have the potential to be an effective knowledge translation tool for the transfer of research results.

Little research has been done examining knowledge users' perspectives on different formats for summarizing research evidence.[9] Despite the growing popularity of infographic formats, little research has examined whether infographics are an effective knowledge translation tool for systematic reviews. The goal of this study was to contribute to this body of research through an initial examination of knowledge user preferences. Our objective was to examine and compare preferences of knowledge users for two different formats of summarizing results from systematic reviews: infographics and critical appraisals.

## Methods

This was a cross-sectional evaluation designed to compare the end user utility and preferences of two data presentation formats: critical appraisals and infographics. The target audience was stakeholders attending the 2013 annual meeting of the National Centre of Excellence in Knowledge Mobilization called TREKK (Translating Emergency Knowledge for Kids). TREKK is a national network of researchers, clinicians, health consumers and relevant organizations with the goal of mobilizing knowledge to improve emergency care for children. TREKK involves 39 general emergency departments across Canada. The purpose of the annual meeting was to update the stakeholders on TREKK activities. Sixty-four stakeholders attended the meeting and 58 participated in viewing the presentation formats and answering a short questionnaire. Stakeholders completed the activity voluntarily; no personal identifying information was collected.

**Fig 1:**
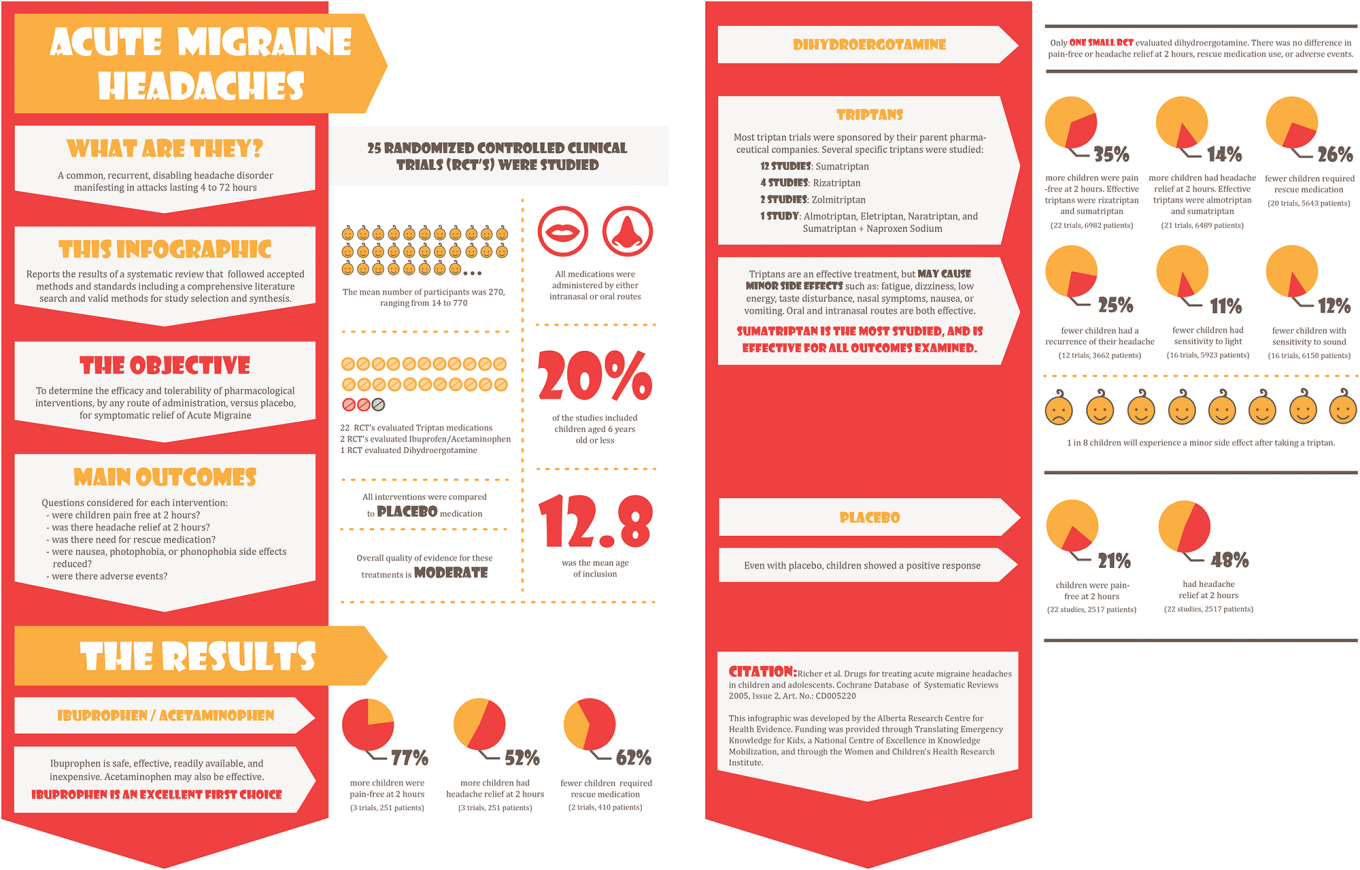
Infographic displaying results of systematic review of drugs for treating acute migraine headaches in children.

**Fig 2:**
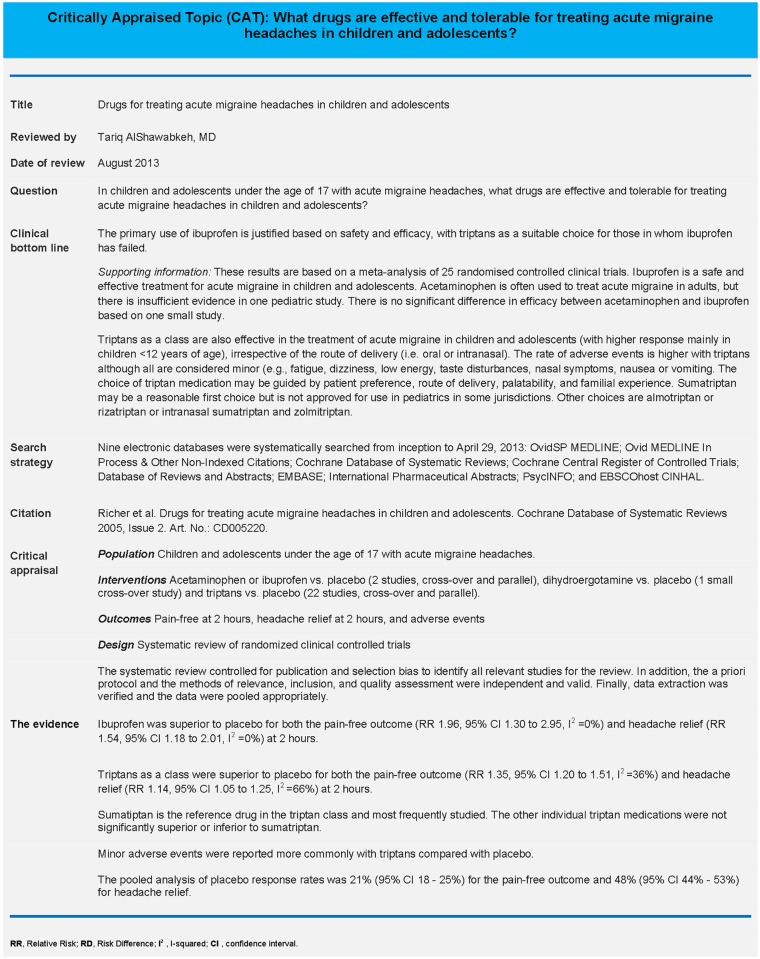
Critical Appraisal of systematic review of drugs for treating acute migraine headaches in children.

We chose to summarize a systematic review of the efficacy and tolerability of different pharmacological therapies for acute migraine headaches in children and adolescents.[15] The topic was selected because it was felt to be of general interest to a range of stakeholders including physicians, nurses, healthcare administrators, and parents. We engaged with a graphic artist to develop an infographic of the chosen review (Fig 1). We also developed a critical appraisal (Fig 2) based on the format used by *Academic Emergency Medicine*. This format was selected based on input from the TREKK steering committee. Information was summarized for the population, interventions, outcomes, study design, and the research evidence from the chosen systematic review. The summary of information followed the PICO format (i.e., population, intervention, comparison, outcome) that is widely accepted for the critical appraisal of evidence.[10]

All stakeholders viewed both of the formats and completed a paper-based questionnaire answering the same set of questions for each of the two formats (S1 File). We compared the formats in terms of the stakeholders' impressions of the clarity, comprehensibility, and aesthetic appeal.[12] These items were assessed using a 10-point Likert scale. We also compared stakeholders' preferences of the two formats, [12] whether they would use the format in their professional role, and for which audiences they believed each of the formats to be most useful. We asked stakeholders about their primary occupational role, and offered them the opportunity to provide additional comments about the two formats.

Differences in clarity, comprehensibility, and aesthetic appeal of the two formats within stakeholders were tested using Wilcoxon matched pairs signed rank test. Wilcoxon signed rank test was also used to compare stakeholders' paired responses to the usefulness of the two formats in their professional role, the usefulness to patients and their caregivers, and for which audiences the two formats would be most appropriate. Mann-Whitney U test was used to compare responses between primary roles. A thematic analysis approach was used to examine text responses to open-ended questions. Data underlying the results presented in this manuscript are available in S2 File.

## Results

A total of 58 stakeholders provided feedback (91%). A range of stakeholders were represented: physicians (n = 27, 48%); nurses (n = 13, 23%); other healthcare professionals, including administrators, allied health professionals, researchers, and research coordinators (n = 9, 16%); and parent advisors (n = 4, 7%). Forty-seven stakeholders reported their preferred format and were divided in their overall preference for the two formats: 24 (51%) preferred the infographic to the critical appraisal (see Table 1).

**Table 1:** Comparison of infographic and critical appraisal formats.

	Infographic	Critical Appraisal	
Characteristics (measured on a 10-point Likert scale)	Mean (SD), n = 57	Mean (SD),n = 56	p-value
Clarity	7.0 (1.9)	7.8 (1.5)	0.03
Comprehensibility	7.1 (1.8)	7.6 (1.5)	0.09
Aesthetic Appeal	7.2 (2.1)	5.0 (2.4)	<0.001
**Appropriateness for different audiences** *	**n (%), n = 56**	**n (%), n = 58**	
Researchers	13 (23.2)	49 (84.5)	<0.001
Other Health Practitioners	32 (57.1)	33 (56.9)	0.98
Public	39 (69.6)	4 (6.9)	<0.001
Media	32 (57.1)	2 (3.5)	<0.001
Decision-Makers	27 (48.2)	34 (58.6)	0.27
Policy Makers	22 (39.3)	31 (53.5)	0.13
Research Funders	14 (25.0)	32 (55.2)	0.001
**Preference by professional role**	**n (%)**	**n (%)**	
All (n = 47)	24 (51.1)	23 (48.9)	0.84
Physicians (n = 22)	7 (31.8)	15 (68.2)	0.02
Nurses (n = 12)	8 (66.7)	4 (33.3)	0.10

* participants were able to select any number of options in terms of the audience for whom either the infographic or critical appraisal would be appropriate

Despite there being no overall preference for either format, preferences of specific aspects of the two formats differed significantly. Stakeholders favored the critical appraisal format in terms of clarity (p = 0.03), whereas for aesthetic appeal, the infographic format was preferred (p<0.001). Stakeholders rated the two formats to be equally comprehensible (p = 0.09). Fifty (87.7%) stakeholders considered the critical appraisal format to be more useful in their professional role than the infographic, although the difference was not statistically significant (p = 0.07). The majority (88%) felt the infographic format would be useful for patients and their caregivers.

There were large differences for which audiences stakeholders believed the two display formats to be most appropriate. The infographic was believed to be most appropriate for the public by 39 (70%) stakeholders (p<0.001) and the media by 32 (57%) stakeholders (p<0.001). The critical appraisal was thought to be more appropriate for researchers (p<0.001) and research funders (p = 0.001). There were no differences in which format was thought to be most appropriate for other health practitioners (p = 0.98), decision-makers (p = 0.27), and policy makers (p = 0.13).

Respondents found the infographic to be visually engaging, attractive, easy to read, and they felt it captured a lot of information. Some respondents found the infographic to be too busy, difficult to interpret and follow, and difficult to determine the take home message. Respondents found the critical appraisal to be clear, directive, professional, and concise; however, they found it more technical and less visually appealing than the infographic.

We compared results for physicians and nurses, the most common primary professional roles represented in our audience. Physicians and nurses responded similarly to all questions except for one: more nurses indicated that the infographic would be useful in their professional role while physicians preferred the critical appraisal (p = 0.04).

## Discussion

Overall, the infographic and critical appraisal formats were equally preferred. Although both formats were well received, the two formats were preferred for different reasons and for different audiences. The critical appraisal format was preferred in terms of clarity and was found to be directive, professional, and concise. The infographic was preferred for aesthetic appeal; it was found to be visually engaging and easy to read while capturing a lot of information. In terms of preferred format for different audiences, it was felt that the infographic was more useful to patients and their caregivers, the public and the media, while the critical appraisal format was believed to be more appropriate for researchers and research funders. Our analyses by professional group showed that nurses preferred the infographic while physicians preferred the critical appraisal. This finding underscores the need to consider the target audience when developing evidence summaries, as well as knowledge translation tools more broadly.

This study is the first of its kind to compare infographic and critical appraisal formats as a knowledge translation tool for the results of systematic reviews. Infographics have become a popular format of data presentation; however, there is little research evidence to support their use or preferences among target audiences.[9] There is a general sense that infographics may be an effective knowledge translation tool because they are aesthetically pleasing and popular. Further, there is evidence in the literature that visual formats are intrinsically more memorable and consistently transferred across people. However, there has been little formal evaluation of the infographic format itself as a knowledge translation tool in the health sciences.[12,14] Formal evaluations of different methods to summarize and disseminate health evidence are needed in order to understand their strengths and limitations, and for which audiences different methods are most appropriate. As demonstrated by this evaluation, there is no 'one fits all' tool. This evaluation involved a single target group and a select systematic review; therefore, results may not be generalizable to other clinical areas or target audiences.

The results of this research demonstrate the importance of understanding the preferences of the target audience when designing knowledge translation tools. Although the critical appraisal and infographic formats were equally preferred overall, there were specific audiences for which each of the two formats was believed to be more appropriate. This information may be helpful to others designing tools to share health information, including evidence from systematic reviews. Further research is needed on the effectiveness of these formats in terms of increasing knowledge and influencing behaviour in order to fully inform the utility of these tools as knowledge translation strategies.

## Supporting Information

S1 File “Infographics Questionnaire” (contains the questionnaire that participants were asked to complete). (DOCX)

S2 File "Infographic Survey Data" (contains original data underlying the results presented in this manuscript). (XLSX)
