# Supplementary material for: Preferences of Knowledge Users for Two Formats of Summarizing Results from Systematic Reviews: Infographics and Critical Appraisals
Source: PLoS One. 2015 Oct 14;10(10):e0140029. doi: 10.1371/journal.pone.0140029 (PMC4605679; doi:10.1371/journal.pone.0140029)
Supplement: S1 File — (DOCX) [file pone.0140029.s001.docx]

| **The following questions are about the red and yellow 2-sided infographic on Acute Migraine Headaches. The first three questions ask you to rate items on a scale from 1 to 10, where 1 is poor and 10 is excellent.** |
| --- |
| **On a scale of 1 to 10, how would you rate clarity? ­­­­__________** |
| **On a scale of 1 to 10, how would you rate comprehensibility? __________** |
| **On a scale of 1 to 10, how would you rate aesthetic appeal? ­­­­__________** |
| **Would you find this form of data presentation useful for you in your professional role (mark circle with an “x”)?**  ○ Yes  ○ No |
| **Do you feel that this form of data presentation would be useful for patients or their caregivers (e.g., parents)?**  ○ Yes  ○ No |
| **For which other audience(s) do you believe this form of data presentation to be appropriate (check all that apply)?**  ○ Researchers  ○ Other health practitioners, specify:  ○ Public  ○ Media  ○ Decision-makers  ○ Policy makers  ○ Research funders |
| **What do you like about this form of data presentation?** |
| **What do you not like about this form of data presentation?** |
| **Do you feel that there is information missing?**  ○ Yes, please specify:  ○ No |
| **Do you have general suggestions for changes in content or layout?** |
| **Please provide any additional comments/feedback:** |

| **What is your primary professional role?**  ○ Physician ○ Nurse ○ Allied health professional ○ Administrator ○ Researcher  ○ Other, please specify: |
| --- |

| **The following questions are about the blue and grey 1-page Critically Appraised Topic on Acute Migraine Headaches. The first three questions ask you to rate items on a scale from 1 to 10, where 1 is poor and 10 is excellent.** |
| --- |
| **On a scale of 1 to 10, how would you rate clarity? ­­­­__________** |
| **On a scale of 1 to 10, how would you rate comprehensibility? __________** |
| **On a scale of 1 to 10, how would you rate aesthetic appeal? ­­­­__________** |
| **Would you find this form of data presentation useful for you in your professional role (mark circle with an “x”)?**  ○ Yes  ○ No |
| **Do you feel that this form of data presentation would be useful for patients or their caregivers (e.g., parents)?**  ○ Yes  ○ No |
| **For which other audience(s) do you believe this form of data presentation to be appropriate (check all that apply)?**  ○ Researchers  ○ Other health practitioners, specify:  ○ Public  ○ Media  ○ Decision-makers  ○ Policy makers  ○ Research funders |
| **What do you like about this form of data presentation?** |
| **What do you not like about this form of data presentation?** |
| **Do you feel that there is information missing?**  ○ Yes, please specify:  ○ No |
| **Do you have general suggestions for changes in content or layout?** |
| **Please provide any additional comments/feedback:** |

| **Overall, which presentation format did you prefer?**  ○ Infographic (red and yellow 2-sided hand-out)  ○ Critically appraised topic (blue and grey 1-sided hand-out) | **If you were to use these, what format would you prefer?**  ○ Paper  ○ Electronic |
| --- | --- |
